# Genetic and phenotypic characteristics of 114 patients with mevalonate kinase deficiency

**DOI:** 10.1186/1546-0096-13-S1-P25

**Published:** 2015-09-28

**Authors:** J Jeyaratnam, N ter Haar, H Lachmann, A Simon, P Brogan, M Doglio, M Cattalini, J Anton, C Modesto, P Quartier, J Frenkel, M Gattorno

**Affiliations:** 1 University Medical Center Utrecht, Eurofever Project, Utrecht, Italy

## Introduction

Mevalonate kinase deficiency (MKD) is a rare autoinflammatory syndrome, characterized by febrile episodes and generalized inflammation.

## Objectives

This study aims to describe the genetic and phenotypic characteristics of MKD in a large international patient cohort.

## Methods

Patients were enrolled in the Eurofever registry (EAHC Project No. 2007332), a registry that retrospectively collects information on patients suffering from periodic fever. An expert on MKD validated all patients on clinical and genetic criteria.

## Results

One hundred and fourteen patients (53 male, 61 female) with two *MVK* mutations were included in this study. The median age of onset was 0.5 years. The median follow up period was 11.5 years. The majority of the patients were Caucasian (90%). Ninety-six patients harboured at least one V377I mutation, fourteen of them had a homozygous V377I mutation. The second most frequent mutation was I268T occurring in 29 patients.

Ninety-nine of 114 patients had recurrent inflammatory episodes, while six patients suffered from a chronic course and nine patients had a chronic course with exacerbations. The median disease duration was five days. The median frequency was 12 per year. Triggers inducing febrile episodes were mentioned in 108 patients, the most important ones were vaccination (n=39), infection (n=18) and stress (n=26).

One hundred and twelve patients suffered from gastrointestinal complaints, most of them suffering from vomiting (69%), diarrhoea (84%) and abdominal pain (88%). Ninety-nine patients also experienced mucocutaneous symptoms, mainly pharyngitis (28%), stomatitis (60%) and maculopapular rash (39%). Seventy-one percent of all patients had arthralgia and 57 percent had myalgia. Arthritis was less common and occurred in 28%. Neurological complaints occurred in 46 patients, most of them suffering from headache (38%). Cerebellar syndrome (3%), mental retardation (4%) and seizures (5%) were noted in some patients. Many patients had constitutional symptoms, such as malaise (65%), weight loss (66%), fatigue (63%) and mood disorders (24%). AA-amyloidosis was noted in six patients. One patient suffered from macrophage activation syndrome, a life-threatening complication characterized by high fever, pancytopenia and liver damage.

Abnormal IgD levels were observed in 55 of 76 tested patients, while 37 of 40 tested patients showed elevated urinary mevalonic acid excretions. Inflammatory parameters, such as erythrocyte sedimentation rates (98%), C-reactive protein (94%) and white blood count (66%), were abnormal in many patients.

## Conclusion

This study describes the clinical and genetic characteristics of 114 MKD patients, which is the largest cohort so far.

